# Juvenile systemic lupus erythematosus onset patterns in Vietnamese children: a descriptive study of 45 children

**DOI:** 10.1186/1546-0096-10-38

**Published:** 2012-11-19

**Authors:** Nguyen Thi Ngoc Dung, Huynh Thoai Loan, Susan Nielsen, Marek Zak, Freddy K Petersen

**Affiliations:** 1 Department of Nephrology, Children’s Hospital 1, 341 Su Van Hanh St., Ho Chi Minh City Dist 10, Vietnam; 2 Paediatric Rheumatology Unit, University Clinic of Paediatrics, Rigshospitalet, Blegdamsvej 9, Copenhagen, DK-2100 Ø, Denmark

## Abstract

**Background:**

Incidence and disease pattern of childhood-onset SLE is reported to differ among ethnic groups.

**Methods:**

To describe disease pattern and 6 month follow-up in a referral based cohort of 45 Vietnamese children with SLE. Forty-five children who were subsequently diagnosed to have systemic lupus erythematosus (f/m = 4/1) were referred to the Ho Chi Minh City Children’s Hospital No.1 during a 12-month period in 2009.

**Results:**

The mean age at diagnosis was 12.8 years (SD = 2.5). Thirty-seven (82%) fulfilled criteria for lupus nephritis (LN). At diagnosis, impressively high SLEDAI and ECLAM scores were recorded (mean and SD), 23.8 (11.6) and 6 (2.3), respectively. The mean renal SLEDAI score was 8.2. The mean haemoglobin (g/dL, SD) was 8.5 (2.1). The Coombs test was positive in 30 of 36 children (83%). The mean plasma creatinine was 0.98 (SD 1.2) and mean Westergren sedimentation rate was 83.6 (SD 37.4). The patient age at diagnosis was positively correlated to the SLEDAI (p = 0.034) and ECLAM (p = 0.022). At 6 month follow-up of the 45 children, 15 patients were in complete remission, 5 were in partial remission, 6 had stable disease, 3 had relapsed, 3 had evolving disease, 2 had ongoing resistant disease and 4 had died. Seven patients were lost to follow-up. A second renal biopsy showed an improved ISN class in 13 of 15; in 2 cases the ISN class remained unchanged.

**Conclusions:**

Forty-five Vietnamese children with SLE were referred to Ho Chi Minh Children’s Hospital No. 1 during a16 month period from 2008–2009. These patients had a strikingly high prevalence of Coombs positive anaemia, a high prevalence of lupus nephritis, and very high SLEDAI and ECLAM scores at the time of diagnosis. While there may be referral biases, our Vietnamese SLE patients appear to have severe disease upon presentation but do reasonably well in the short-term.

## Background

Fifteen to twenty percent of all systemic lupus erythematosus (SLE) patients have a disease onset before the age of 16 years. Childhood onset SLE is an unusual autoimmune disease with high risk of severe morbidity and mortality [1]. Incidence and disease patterns do differ among ethnic groups. It has been established that non-Caucasians have a higher incidence than Caucasians, and that renal disease is more frequent in non-Caucasian SLE children. Disease activity and damage, however, are primarily associated with major organ disease independent of the patient's ethnicity [2-4]. The objective of the current study was to investigate disease patterns at the time of diagnosis in a referral-based consecutive cohort of 45 Vietnamese children diagnosed with SLE at Ho Chi Minh Children’s Hospital No. 1 and to perform a 6-month follow-up with emphasis on mortality and lupus nephritis (LN).

## Methods

### Diagnosis

Forty-five consecutive Vietnamese children with SLE participated in this prospective registration study. All new cases of childhood-onset SLE referred during the period from July 2008 to November 2009 to the tertiary Ho Chi Minh City (HMC) Children’s Hospital No. 1 were included. The children were referred either from rural provinces, from the southern part of Vietnam, or from local hospitals in HMC. The American College of Rheumatology (ACR) SLE criteria were used for the diagnosis [5]. At the time of diagnosis, symptoms and biochemical parameters were recorded and used for SLE disease activity scores. These included the SLE Disease Activity Index (SLEDAI) and European Consensus Lupus Activity Measurement (ECLAM) [6-8].

The SLEDAI is a validated disease activity measure for childhood onset SLE with a total score of 0 – 105. The tool consists of 24 weighted items grouped into following 9 domains: central nervous system (CNS), vascular, renal, musculoskeletal, serosal, dermal, immunologic, constitutional and hematologic. Renal disease activity was measured by the SLEDAI renal domain items [1]. ECLAM consists of 34 items grouped into 12 domains (9 clinical manifestations and 3 laboratory assessments) and measures active and evolving disease activity. ECLAM scores ranges from 0 to 10.

All the SLEDAI and ECLAM scoring was done by one author (ND). Renal biopsies at diagnosis were done in suspicion of severe nephritis and evaluated according to the ISN (International Society of Nephrology) lupus nephritis grading system by an independent pathologist [9]. A follow-up biopsy after initial treatment was done whenever possible.

The following clinical symptoms and parameters were recorded: general symptoms (fatigue, pain and discomfort, general malaise), fever, malar rash, discoid rash, photosensitivity, alopecia, mouth ulcerations, vasculitis, nephritis, CNS symptoms (headache, seizures, psychosis, visual symptoms, cranial nerve manifestations and other possible neurological manifestations), myositis, serositis, gastrointestinal symptoms and pulmonary symptoms.

The blood tests performed were: haemoglobin, white blood cell count (WBC) and differential count, platelet count, erythrocyte sedimentation rate (ESR), C-reactive protein, serum creatinine, blood urea nitrogen, albumen, a 24-hour urinary protein excretion, red and white casts in urine, lactate dehydrogenase, creatinine kinase, glutamic-oxaloacetic transaminase, and thyroid parameters. Other tests more specific for SLE were done including a Coombs test, C3 and C4 complement levels, the antinuclear antibodies (ANA), anti-DNA, and the anticardiolipin antibodies IgG and IgM. The ANA and anti-DNA levels were assessed using ELISA. Furthermore, blood pressure, heart rate, height, and weight were recorded. The local scientific committee approved the study.

### Treatment

Induction treatment was administered according to the local guidelines, which were as follows.

1) All cases with CNS involvement or lupus nephritis ISN class IV or class III (with any symptoms: nephrotic syndrome, hypertension, elevated serum creatinine concentration, renal biopsy with 40 to 50 percent of glomeruli affected and/or crescent) were treated with:

a) Intravenous methylprednisolone (1 g/1.73 m2/day x 3–6 days, with a maximum dose of 1 g per treatment) and monthly intravenous

b) Intravenous cyclophosphamide (Cyc) (1 g/m2/month; maximum single Cyc dose 0.5 g) x 6 consecutive months;

c) Oral prednisolone 1–2 mg/kg/day reduced according to disease activity.

2) Maintenance therapy consisted of azathioprine (1–2 mg/kg/day) and oral prednisolone. Additional treatment with an ACE inhibitor was administered to the majority of nephritis patients. All other cases or cases with mild nephritis (Class I, II, mild III) were treated with systemic steroid only.

### Renal outcome measures

A renal biopsy at diagnosis was done if proteinuria exceeded 1 g/24 hours. Biopsy at 6 month followup was performed only in the nephritis cases treated with cyclophosphamide.

Renal outcome measures was in accordance with the local clinical practice classified as:

1) Complete remission (CR): If presence of normal serum creatinine, improvement in C3 level, proteinuria < 100 mg/m2/day or < 1+ on urinary dipstick and/or inactive urinary sediment;

2) Partial remission (PR): If stabilization or improvement in serum creatinine level, improvement in C3 level, persistent reduction of proteinuria or, if in the nephrotic range at baseline, a decrease > 50% decrease in proteinuria and renal protein excretion <50 mg/kg/day, if in the non-nephrotic range at baseline, a decrease < 50% of the pre-treatment value but renal protein excretion not more than 100 mg/m2/day, and reduction in active urinary casts;

3) Nonresponse (NR) If deterioration of renal function occurs exclusive of other causes, increase in proteinuria or reduction in proteinuria but not to the extent of CR or PR. At the 6 month follow-up CR or PR was regarded as favourable outcome.

4) A poor outcome was present in case of stable disease (SD), resistant disease (RD), evolving disease (ED), relapse (RE) or death (MO).

## Results

The mean age at SLE diagnosis was 12.8 years (SD = 2.5). The female/male ratio was 4/1. All children were of Vietnamese origin. Table 1 displays demographics and disease characteristics at the onset of the SLE. The mean interval from initial symptoms to the SLE diagnosis was 23 weeks (SD = 58). Upon exclusion of 3 outlier patients, the interval was reduced to a mean of 7.8 weeks (SD = 9).

**Table 1:** Demographics and characteristic at disease onset in 45 Vietnamese children with SLE

**Parameter**	**Value**	
Number of patients	45	
Female: male	4:1	
Median age at diagnosis	12.1 years	
Disease characteristics at diagnosis	Present n (%)	Absent n
Anti-DNA	43 (95)	2
Nephritis	37 (82)	8
Hematology	35 (78)	10
Malar rash	30 (67)	15
ANA	30 (67)	15
Arthritis	26 (58)	19
Photosensitivity	24 (53)	21
Ulcers	17 (38)	28
Serositis	16 (36)	29
CNS	7 (16)	38
Discoid changes	6 (13)	39
Renal biopsy	30 (67)	15

The most frequent initial SLE symptoms were: rash, fever, edema and arthritis. The group as a whole had a mean SLEDAI and ECLAM scores of 23.8 (SD = 11.6) and 6 (SD = 2.3), respectively, and a mean renal SLEDAI score of 8.2. Thirty-three of the 45 children fulfilled the criteria for lupus nephritis (LN), of which 29 had a renal biopsy. ISN Class IV was found in 20 cases and ISN Class III in 8 cases, one case had complex presentation with ISN Class III and V simultaneously. Nephritis was more prevalent in the children < 12 years of age at the time of the SLE diagnosis. This finding was, however, not statistically significant (Fisher’s Exact Test p = 0.06). Seventy percent of the children had a serum complement (C3 or C4) level below normal. C3 was below normal in 42% and C4 was below normal in 70% of the LN cases.

Elevated blood pressures were found in 20 of 45 patients primarily in LN patients. Hypertension was defined as systolic and/or diastolic blood pressure above the 95th percentile for gender and age. CNS involvement was found in 6 cases (13%). Serositis was found in 16 (36%) cases. Haemolytic anaemia with a positive Coombs test was found in 12%. Overall, haematological abnormalities were found in 81% of the children at presentation. Forty-three patients (96%) had an elevated antidsDNA. Mean erythrocyte sedimentation rate (ESR) was 83.6 (SD = 37.4, normal 0–20 mm/hour).

At a 6-month follow-up of the 33 renal patients, 15 patients were in CR, 5 in PR, 6 had SD, 3 had ED, 2 had RD, 3 had RE and 4 MO. Seven patients were lost to follow-up. Fifteen had a second renal biopsy after the course of 6 Cyc treatments. In 13 cases the ISN class had improved and in 2 cases it remained unchanged. In the first six months, 4 of the original 45 patients were know to have died, 2 before initiating treatment (1 with circulatory collapse and 1 of undiagnosed pneumonia), and 2 of tuberculosis shortly after initiating Cyc treatment.

**Figure 1:**
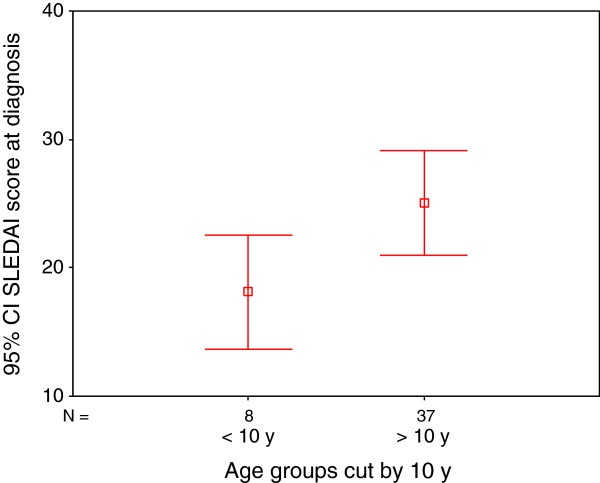
**Mean and 95% confidence intervals (CI) SLEDAI scores according to age in 45 Vietnamese children with SLE.** The cut-off is 10 years of age. p = 0.018.

Baseline parameters from the time of the diagnosis were investigated in order to look for predictors of short-term disease outcome. A low neutrophil count at the time of the diagnosis was associated with poor disease outcome (p = 0.01). The patient age at the time of the diagnosis was positively correlated to the SLEDAI (p = 0.034) and ECLAM (p = 0.022), which was attributed to a wider range of organ involvement in the oldest children (Figure 1).

## Discussion

In a prospective study, all new cases of SLE referred to HCMC Children’s Hospital No. 1 during a 16 months period were investigated. The ratio female/male of 4/1 was in accordance with the literature. However, a number of findings differed somewhat from previously published reports on childhood-onset SLE. First, the relatively high mean age of 12.8 years at the time of the diagnosis was unusual. This finding could be explained by the fact that no children below the age of 5 years were included, what could be a true pattern but is more likely a result of underreporting/lack of diagnosis. An Italian group found that young children below 2 years of age at onset had very unspecific symptoms as rash and fever, making diagnosis more difficult [10]. In Vietnam, where infectious diseases are one of the major reasons of morbidity and mortality in young children, the diagnosis of SLE might prove particularly difficult. The mean age at diagnosis of childhood-onset SLE has been described to be different among ethnic groups, and is reported to be lower among non-Caucasian versus Caucasians [3,4,11-13].

SLEDAI and ECLAM have been validated as measures of disease activity for evaluation of response to therapy in SLE. ECLAM seems to be slightly superior to SLEDAI in measuring change over time, while SLEDAI seems to be slightly superior to ECLAM in measuring accumulated disease activity as a predictor of damage [6-8]. In general, at the time of referral children often have more severe disease activity with higher SLEDAI scores than adults [1,10,14]. The disease patterns for the current study showed remarkably sick children with a mean SLEDAI of 23.8 and a moderately high SLEDAI renal score. It remains to be investigated whether SLEDAI and/or ECLAM exhibit sufficient construct validity, accuracy and responsiveness in follow-up studies of SLE.

One of the major contributors to the high SLEDAI score was the 75% proportion of LN cases. This finding was in accordance with the literature [1,3,10,12,13]. All LN cases fulfilled criteria of nephritis according to the SLE criteria and biopsy was done in 89%, the remaining not being suspected of having severe nephritis. As described by others, class IV was the most dominant feature on biopsy [1,3,10,11,14-18]. Of all biopsies from nephritis cases, 61% were classified as ISN class IV and 24% as ISN class III. No cases of class I - II was found in the initial biopsy, which could be explained by the fact that biopsies were done in suspected severe SLE nephritis cases only.

Hypertension was found in 20 of 45. Hypertension at the time of the diagnosis was not predictive of the short-term renal outcome. All class IV cases were treated according to the same protocol including induction therapy with low-dose Cyc and systemic steroids in 6 consecutive infusions 1 month apart. Follow-up biopsy (n = 13) after 6 months showed marked improvement in 11 from class IV to III (n = 7) and class II (n = 4), in 2 slightly improvement among class III. Normalization of the C3 and C4 complements and reduction of proteinuria by greater than 25% by week 8 has been showed to strongly predict a good renal response to the SLE treatment [19]. Despite our cohort having a very high renal SLEDAI score at diagnosis, the majority of the nephritis patients showed significant improvement at 6 months followup. The short follow-up did not allow prediction of renal survival time, although the intensive treatment might prove predictive of a favourable renal outcome in comparison with results from other studies [11,16-18,20]. As childhood onset SLE is a chronic disease with exposure to lupus flares over many years, it will be interesting to see if there is a prognostic benefit to this early response to therapy in our renal patients in an extended prospective followup.

The most remarkable haematogical finding was a very high percentage of Coomb’s positivity (83%) with or without haemolyctic anaemia. The laboratory technique used was the slide rather than the tube method. The technique is well standardized and unlikely to yield false positive results. An explanation for this phenomenon could be the high incidence of infections in Vietnamese children, which in some instances may be followed by a positive Coombs test.

Central nervous system disease, defined as neurological involvement plus seizures and psychosis, was found in 13% of cases. Others have described an incidence of CNS disease from 7.7% to 45%, with infantile SLE having the highest incidence and a trend of a greater incidence of CNS involvement in Afro-Americans than in Asian [10,14,20,21].

Cardio-pulmonary disease was detected in 35% of the children, which is in accordance with the lupus literature, except for the Italian group who found a very high incidence in infantile SLE (approximately 75%), and for Gedalia et al., who found a higher incidence in Afro-Americans compared to Latin-Americans [10,12,14,20,22].

Malar rash was observed in 66% and discoid lupus in 13%, what was somewhat different from the literature and may be different among ethnic groups [10,12,14,18,20,22]. Gedalia et al. found a higher frequency of malar rash and discoid rash among Afro-Americans than Latino-Americans. Our figures resemble what was found in Afro-Americans [22].

The very high percentage of positive anti-DNA (95%) is a well known feature of SLE. Very few investigations of anti-phospholipid antibodies were done (N = 10), 3 IgM positive; 8 IgG positive. No coagulation disturbances were detected. Almost none had investigation of thyroid hormones due to economic reasons.

One patient died prior to treatment in a picture of circulatory collapse shortly after diagnosis without evidence of a bleeding disorder or intravascular dehydration. This may have been a case of SLE carditis, though this is speculative. Jakes et al. found deaths due to cardiovascular involvement in 6-40% in the Asia-Pacific region [3].

One child died after a severe pneumonitis, with no microorganism identified, perhaps due to SLE lung involvement. Two patients died of TB. Both were diagnosed with pulmonary TB after the first infusion of cyclophosphamide and were on high dose corticosteroids. Chest x-ray before treatment had been normal. However, no other TB testing was done.

Only a few studies exist on the incidence and prevalence of SLE in South-East Asia. In the available literature from the region, the reported incidence rates range from 0.9 to 3.1 per 100.000 children [3]. The population in Southern Vietnam below 16 years of age is estimated to be approximately 10 million. Assuming that all new childhood SLE patients from South Vietnam were referred to HCM Children’s Hospital No. 1 during the study period, a minimum incidence of approximately 0.4/100,000 children might be calculated. However, the paediatricians at HMC Children’s Hospital most likely only get to see the top of the iceberg. The true incidence and prevalence may be considerably higher for several reasons including under-reporting of the disease, the diagnostic challenges of SLE, and selection bias.

## Conclusions

We report a cohort of 45 newly diagnosed SLE children from the Southern part of Vietnam. The disease pattern resembles in many ways the clinical patterns described by others of childhood onset SLE. However, the cohort did differ by demonstrating a very severe disease activity with very high SLEDAI scores and high SLEDAI renal scores at diagnosis. Although the short time follow-up of 6 months showed favourable histological treatment response, the data do not allow prediction of long time patient and renal survival. We plan an additional follow-up study that might answer some of these questions.

## Competing interests

The authors declare that they have no competing interests.

## Authors’ contribution

All authors read and approved the final manuscript. NTND has participated in data collection and writing of the paper, HTL has participated in data collection and writing of the paper, NS has participated in statistical analysis and writing of the paper, ZM participated in statistical analysis and writing of the paper, PFK participated with the idea behind the study and writing of the paper.
